# Inflammasomes and the IL-1 Family in Bone Homeostasis and Disease

**DOI:** 10.1007/s11914-022-00729-8

**Published:** 2022-05-14

**Authors:** Hsu-Wen Tseng, Selwin Gabriel Samuel, Kate Schroder, Jean-Pierre Lévesque, Kylie A Alexander

**Affiliations:** 1 Mater Research Institute, Translational Research Institute, The University of Queensland, 37 Kent Street, Woolloongabba, QLD Australia; 2 Institute for Molecular Bioscience, The University of Queensland, St Lucia, QLD Australia

**Keywords:** Inflammasome, Interleukin-1, Interleukin-18, NLRP3, Bone

## Abstract

**Purpose of Review:**

Inflammasomes are multimeric protein structures with crucial roles in host responses against infections and injuries. The importance of inflammasome activation goes beyond host defense as a dysregulated inflammasome and subsequent secretion of IL-1 family members is believed to be involved in the pathogenesis of various diseases, some of which also produce skeletal manifestations. The purpose of this review is to summarize recent developments in the understanding of inflammasome regulation and IL-1 family members in bone physiology and pathology and current therapeutics will be discussed.

**Recent Findings:**

Small animal models have been vital to help understand how the inflammasome regulates bone dynamics. Animal models with gain or loss of function in various inflammasome components or IL-1 family signaling have illustrated how these systems can impact numerous bone pathologies and have been utilized to test new inflammasome therapeutics.

**Summary:**

It is increasingly clear that a tightly regulated inflammasome is required not only for host defense but for skeletal homeostasis, as a dysregulated inflammasome is linked to diseases of pathological bone accrual and loss. Given the complexities of inflammasome activation and redundancies in IL-1 activation and secretion, targeting these pathways is at times challenging. Ongoing research into inflammasome-mediated mechanisms will allow the development of new therapeutics for inflammasome/IL-1 diseases.

## Introduction

### The Inflammasome and IL-1 Family

The innate immune system provides the first line of defense against pathogens due to its ability to recognize a wide range of pathogens through germline-encoded receptors called pattern recognition receptors (PRRs). PRRs are expressed by numerous myeloid cell types including macrophages, monocytes, osteoclasts, dendritic cells, neutrophils, as well as non-myeloid cell types such as epithelial, endothelial, and mesenchymal cells. When activated, PRRs rapidly trigger downstream signaling pathways which lead to inflammatory responses. PRRs recognize conserved microbial molecular structures termed pathogen-associated molecular patterns (PAMPs) as well as danger-associated molecular patterns (DAMPs) released by injured cells or tissues.

The interleukin-1 (IL-1) family of cytokines is primarily associated with innate immunity and affects a broad spectrum of inflammatory and immune responses. Certain members of the IL-1 family (IL-1β and IL-18) require caspase-mediated proteolytic cleavage for functional activation [1]. The inflammasome is a macromolecular structure that assembles in the cytosol of cells in response to infection (via PAMPs) or tissue injury (via DAMPs) and leads to the activation of inflammatory caspases (caspase-1 and -11 in mouse, and -1, -4, and -5 in humans) [2]. Active caspase-1 cleaves pro-IL-1β and pro-IL-18 into their biologically active forms [3, 4]. Gasdermin D (GSDMD) is also cleaved and activated by inflammatory caspases, which leads to the assembly of GSDMD pores in the plasma membrane, enabling the release of active IL-1β and IL-18 into the extracellular milieu [5].

**Fig. 1:**
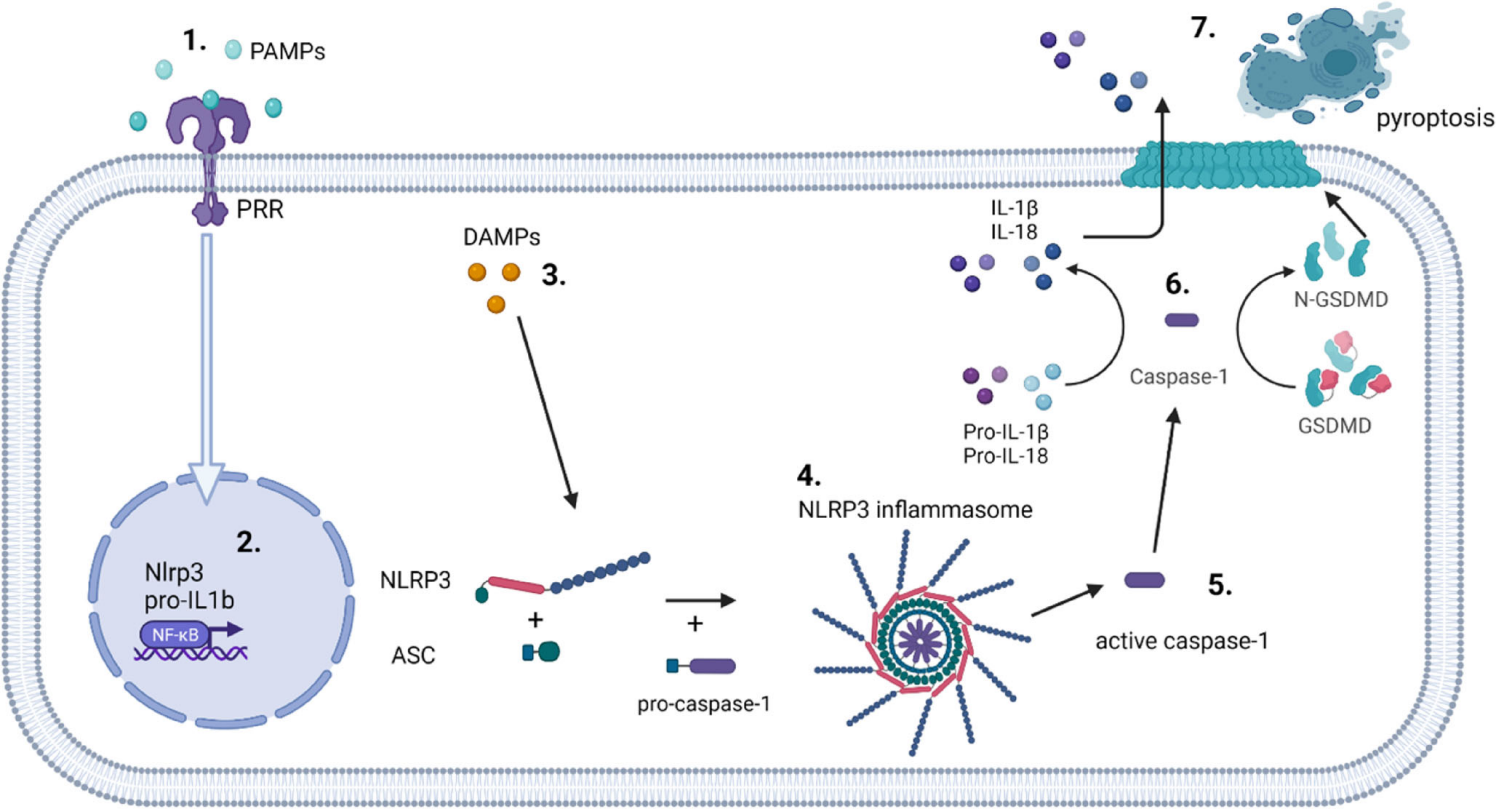
Assembly of the NLRP3 inflammasome. Following a priming signal, e.g., recognition of a PAMPs by PRRs (1) the transcription of *Nlrp3* and *Il1b* genes are activated in a NF-κB-dependent manner (2). In parallel, an activation signal is provided by a variety of stimuli (DAMPs, e.g., ATP, K^+^ ionophores) (3), all of which lead to activation of the NLRP3 sensor protein, NLRP3 oligomerization and subsequent association with the ASC adaptor subunit, which forms a large ASC-containing speck with subsequent docking of pro-caspase-1 (4), where pro-caspase-1 dimerization and auto-catalysis produces active caspase-1 (5), which then cleaves and activates pro-IL1β, pro-IL-18 and GSDMD (6). Cleaved GSDMD forms pores within the membrane which enables release of mature IL-1β and IL-18 from the cell and triggers pyroptosis (6). Other canonical inflammasomes function in a similar manner but with different sensor proteins. Created with BioRender.com

Multiple types of inflammasomes have been identified [3, 6], and can be divided into two broad categories: (1) canonical, which activate caspase-1, and (2) noncanonical, which activate caspase-11 in mouse and caspase-4/5 in human. Canonical inflammasome signaling first requires a licensing step (also known as priming), in which cytokines (e.g., TNF, IL-1) or PAMPs upregulate the expression of pro-IL-1β, and in some cases the inflammasome sensor molecule (e.g., NLRP3) (Fig. 1). Inflammasome assembly requires a second signal (e.g., DAMP recognition by an inflammasome sensor), which results in inflammasome sensor activation and oligomerisation. Next, there is recruitment of the inflammasome adaptor protein (e.g., ASC; apoptosis-associated speck-like protein containing a CARD (caspase recruitment domain), encoded by the *Pycard* gene) (Fig. 1). Once the sensor protein oligomerizes and interacts with ASC, they form a single macroscopic speck in the cytosol. This speck subsequently docks pro-caspase-1 monomers triggering caspase-1 activation by dimerization and autocatalysis [4] (Fig. 1). Active caspase-1 then cleaves pro-IL-1β and pro-IL-18 into biologically active cytokines. The final step is the release of mature IL-1β and IL-18 from the cytosol into the extracellular fluid, which occurs as a consequence of passage of these cytokines through GSDMD pores across the plasma membrane [7], and drives a particular form of cell death called pyroptosis (Fig. 1). In non-canonical inflammasome signaling, caspases are able to directly bind intracellular lipopolysaccharide from Gram-negative bacteria and can directly mediate GSDMD cleavage and pyroptosis, as well as trigger a secondary activation of the canonical inflammasome [8].

Not all IL-1 family members require cleavage via inflammatory caspases. For example, IL-1α is constitutively expressed as pro-IL-1α, which is biologically active but can be further activated by inflammasome-independent proteolytic cleavage. Inflammatory stimuli also enhance the transcription of the *Il1a* gene. Pro-IL1α binds to the plasma membrane of phagocytes and can be further activated and released via protease-mediated cleavage by chymase, granzyme B, or neutrophil elastase, or be released in a soluble form [9]. Pro-IL-33 and pro-IL-37 are also biologically active [10]. A hallmark of IL-1 signaling is the redundancy of IL-1 family members capable of binding the same cognate receptor. For example, IL1R1 binds IL-1α, IL-1β, and their endogenous antagonist IL-1RA albeit with different affinities [11]. IL-1 signaling can be controlled via the endogenous IL-1 antagonist IL1RA, that competes for the receptor as a negative feedback inhibitor of excessive IL-1 signaling [1].

IL-18 forms a complex with the IL-18 receptor α and β chain (IL-18Rα and IL-18Rβ) which activates downstream signaling. IL-18 signaling is regulated by IL-18 binding protein (IL-18 BP), a circulating endogenous IL-18 antagonist with high affinity for IL-18 [12]. IL-37 exerts an anti-inflammatory effect by binding to IL-18Rα, but recruits IL-1R8 instead of IL-18 Rβ [13].

It is clear that inflammasome signaling is vital for rapid responses to infection and injury; however, inflammasome dysregulation and aberrant IL-1 expression also drives the initiation and progression of arthrosclerosis [14], type 2 diabetes and obesity [15], neurodegenerative diseases [16], and cancer [17]. In this review, we further discuss functions for inflammasomes and IL-1 family activity in bone homeostasis as well as in pathologies of bone accrual and loss, as well as current and future therapies for these diseases.

## Inflammasomes and IL-1 in Physiological Bone Development

It is well established that the skeletal system can be impacted by chronic inflammation but less appreciated is the role that the inflammasome and IL-1 plays in physiological bone development.

Primary murine osteoblasts express NLRP3, ASC, and caspase-1 mRNA and proteins [18]. Osteoblasts present on bone surfaces also express NLRP3 protein [19]. While numerous commercially available *Nlrp3*^−/−^ strains do not report bone phenotypes, one study established in 4-week-old male and female *Nlrp3*^−/−^ mice display impaired skeletal development with reduced full body length, lower trabecular bone volume, lower trabecular thickness and number, as well as a deficient growth plate [19]. NLRP3 expression has also been observed in hypertrophic chondrocytes, and *Nlrp3*^−/−^ mice display reduced bone sialoprotein expression in hypertrophic chondrocytes [19], suggestive of a role for NLRP3 during chondrocyte maturation. In vitro generated osteoclasts also express NLRP3 and NLRC4 (NLR CARD domain containing-4, an adaptor for the NLRC4 inflammasome) when exposed to LPS and nigericin [20] and the loss of NLRP3 or NLRC4 inflammasomes in mice attenuated osteoclast differentiation in vitro. Interestingly, this study by Alippe and colleagues observed that male *Nlrp3*^−/−^ mice (but not *Nlrc4*^−/−^) at 8 months of age, displayed a significantly higher bone mass and bone mineral density compared to wild-type counterparts [20]. This observation is in stark contrast to the study by Detzen at al. mentioned above [19]. These contrasting conclusions highlight the complexities of using small animal models where analysis of bone phenotypes at different ages (4 weeks versus 8 months) or different sex may potentially provide explanations for these phenotypic discrepancies.

IL-1 was originally identified as ‘osteoclast-activating factor.’ IL-1 has been proposed to stimulate osteoclastogenesis under physiological conditions indirectly by stimulating prostaglandin E2 synthesis in osteoblasts [21] as well as increasing the expression of receptor activator of NF-κB ligand (RANKL) expression in osteoblasts [22]. In vitro studies also confirmed that IL-1 directly promotes osteoclast formation, multinucleation, pit forming activity [23], as well as survival [24, 25]. Mice administered IL-1β develop hypercalcemia and display increased osteoclast numbers, and treatment with chimeric osteoprotegerin (OPG; a RANKL antagonist) reduced hypercalcemia and osteoclast number [26]. IL-18 is expressed by osteoblasts [27] and chondrocytes [28], IL-18 also inhibits osteoclastic bone resorption indirectly [27] and upregulates OPG in stromal/osteoblast cells [29]. Small animal models have provided further insights into how the IL-1 family cytokines influence bone development. Under germ-free conditions, *Il1a*^−/−^, *Il1b*^−/−^, or *Il1a*^−/−^ x *Il1b*^−/−^ mice have increased bone mass (femur mineral density, trabecular bone mass, and cortical thickness) compared to wild-type controls [22], suggestive of roles in physiological bone metabolism. In addition, mice defective for the *Il1r1* gene exhibit a narrow growth plate, due to a smaller hypertrophic zone [30], suggestive of a role in hypertrophic chondrocyte differentiation. However, these observations are in contrast to another study finding no changes in bone mass in *Il1r1*^−/−^ mice, concluding that normal bone development can occur in the absence of IL-1R1 [31]. These contrasting conclusions highlight the complexities of small animal models with differences in mouse genetic backgrounds, housing conditions, and bone mass measurement methods all being suggested as the reasons behind these discrepancies. Finally, transgenic mice that overexpress IL-18 were shown to have decreased trabecular bone turnover and altered cortical structure [32]; however, no bone phenotype has been reported for *Il18*^−/−^ mice.

Overall, a few studies have linked the inflammasome and IL-1 family members to bone development, but to date, most research has focused on inflammasome regulation for prevention of disease, which is further discussed in sections below.

## Inflammasomopathies with Skeletal Phenotypes

The term ‘inflammasomopathies’ describes a group of mechanistically similar diseases which have inappropriate inflammasome activation. The diseases with impacts on the skeleton are further discussed below.

### Cryopyrin-Associated Periodic Syndromes

Cryopyrin-associated periodic syndromes (CAPS) are autoinflammatory disorders mediated by dysfunctional inflammasome activation caused by heterozygous germline or somatic gain-of-function mutations in the *NLRP3* gene [33, 34]. These mutations lead to a hyperactive NLRP3 inflammasome, increased activation, and cleavage of caspase-1 and subsequent IL-1β and IL-18 cleavage, activation, and secretion. Clinically, CAPS refers to three disorders (listed in order of increasing disease severity): familial cold autoinflammatory syndrome (FCAS), Muckle-Wells syndrome (MWS), and neonatal-onset multisystem inflammatory disease (NOMID), all with shared and unique clinical features. CAPS diagnosis can be challenging, as patients show a heterogeneous multi-system clinical presentation. CAPS is frequently characterized by cutaneous, neurological, ocular, and otologic features. Numerous studies have also established that CAPS patients display varying (20–90%) levels of musculoskeletal manifestations such as arthralgia, arthritis, and myalgia, with more severe skeletal abnormalities such as patellar overgrowth, bone deformity, bone/joint erosion, and/or osteolytic lesions [35–37] (Table 1). NOMID is the most severe form of CAPS. NOMID arthropathy begins in infancy [33] with increased patellar and epiphyseal long bone ossification, and development of calcified masses that deform the adjacent metaphysis and epiphysis [35]. These skeletal deformities are a common cause of disability in NOMID patients. NLRP3 is not only expressed in monocytes and neutrophils but also in human chondrocytes, accounting for the arthropathy seen in these syndromes [34].

**Table 1:** Summary of skeletal manifestations in CAPS patients

	Skeletal manifestations in CAPS		
	FCAS (mild phenotype)	MWS (intermediate phenotype)	NOMID (severe phenotype)
Disease onset	<6 months–adulthood	Early childhood–adulthood	Perinatal
Musculoskeletal manifestations	Myalgia, arthralgia [38, 39]	Myalgia, arthralgia and oligoarthritis [38, 39]	Myalgia, arthralgia, arthritis, patellar and long bone overgrowth, abnormal epiphyseal and metaphyseal calcification, bone and joint deformity, bone/joint erosion, osteolytic lesions [33–37]

In order to further understand CAPS disease pathogenesis, gain-of-function small animal models were developed [40–42]. One group developed two models, each with Cre recombinase inducible mutations *Nlrp3*^A350V^ and *Nlrp3*^L351P^, corresponding to the human mutations *NLRP3*^A352V^ and *NLRP3*^L353P^ [41], which are associated with MWS and FCAS. A NOMID mouse model was also developed which contains a knock-in for the *Nlrp3*^D301N^ mutation (ortholog of *NLRP3*^D303N^ in humans), a mutation associated with NOMID patients [40]. These mouse models replicate clinical and pathologic features seen in humans and have arthropathy such as growth retardation and osteopenia; however, both models demonstrated early mortality. These models have confirmed a pivotal, but not exclusive, role for IL-1β in CAPS [41], whereby treatment with the small synthetic NLRP3 inflammasome inhibitor MCC950 effectively and specifically blocked NLRP3 activation in vivo with increased survival and body weight [43]. Neutrophils have been suggested as a cellular driver of CAPS pathology [44]. Gasdermin D deficiency has also been demonstrated to reduce NOMID pathogenesis in mice [45•], which supports discovery efforts aimed at identifying selective gasdermin D inhibitors.

IL-1 blockade has been the standard of care for CAPS patients. Specifically, Anakinra (a recombinant human IL-1 receptor antagonist) [46], Rilonacept (a recombinant fusion protein consisting of the extracellular domain of IL-1 receptor fused with IgG1 Fc region; IL-1 trap) [47], and Canakinumab (a humanized neutralizing IL-1β monoclonal antibody) have all been trialed [48]. Treatment resulted in reduced inflammatory symptoms such as rash severity, conjunctivitis, as well as reduced C-reactive protein (CRP) and serum amyloid A (SAA) levels, and over time, reduced organ inflammation [49, 50]. However, a consensus has not been reached on whether IL-1 antagonist therapies are routinely successful in reducing CAPS skeletal manifestations. There are studies that did not report positive outcomes [51]. However, there are a few studies indicating that IL-1 antagonist therapies did result in beneficial skeletal outcomes but these are only in a few patients. For example, one study concluded that anakinra treatment (1 mg/kg/day) had no influence on clinical manifestations not closely related to ‘inflammation’ such as bone dysplasia. In this study, 4 patients with growth failure present at baseline showed no growth catch up after treatment. However, in one patient, there was a dramatic clinical and radiologic improvement in a chodroblastoma after 2 years of anakinra [52]. An additional study, similarly, showed an improvement in arthropathy in one NOMID infant after anakinra treatment. Radiological examination confirmed metaphyseal fraying and cupping and widening of the growth plate in the distal femur at baseline, with follow-up radiographs indicating a reduction in growth plate widening and longitudinal femur and tibia growth after 20 months of observation [53]. Another group reported CAPS patients at baseline, displayed subnormal BMD values (mean (SD) Z-scores of − 2.22 (0.51), − 1.96 (1.39), − 1.92 (1.92), and − 1.75 (1.84) at the femoral neck, L1–L4, total hip and intertrochanteric, respectively) and after 60 months of anakinra the mean Z scores increased (− 1.08 (1.63), − 0.74 (1.47), − 0.80 (1.60), and − 0.96 (1.63) at the femoral neck, L1–L4, total hip, and intertrochanteric, respectively) [54]. More recently, a longer-term study was able to illustrate some resolution of arthropathy after anakinra in some but again not all patients [55]. Clearly, larger studies are necessary to further assess whether IL-1 antagonist therapies provide significant improvement in the bone pathologies observed in CAPS patients.

CAPS patients also display elevated levels of ASC and mature IL-1β in peripheral blood mononuclear cells and treatment of mononuclear cells ex vivo with MCC950 reduced mature IL-1β production, highlighting the therapeutic potential of NLRP3 small molecule inhibitors [56•]. As deubiquitination is essential for efficient NLRP3 inflammasome activity, blocking deubiquitination prevented IL-1β production and inhibited the activation of multiple mouse NLRP3 mutants linked with CAPS [57•], which were previously shown not to benefit from MCC950 treatment [58]. This also suggests that posttranslational modification of NLRP3 is an additional future pharmacological target for CAPS.

Other treatments such as non-steroidal anti-inflammatory drugs, antihistamines, and immunosuppressants were trialed with disappointing results [59].

### Deficiency in IL-1 Receptor Antagonist

Deficiency in IL-1 receptor antagonist (DIRA) is a rare autosomal recessive autoinflammatory disease in which a deletion in the *IL1RN* gene (which encodes IL-1RA) results in a loss of production of IL-1RA [60]. As IL-1RA is a natural endogenous inhibitor of IL-1 [61], DIRA infants have unopposed stimulation of IL1R1 [60]. DIRA has disease phenotypes similar to CAPS, where neonates present with skin and bone manifestations. Over time, DIRA patients present with an array of skeletal abnormalities such as ballooning of long bones and anterior rib ends, multifocal osteolytic lesions, widening of clavicles, metaphyseal erosions, and heterotopic ossifications at the proximal end of femurs [60, 62]. DIRA has been studied in small animal models using mice defective for IL-1RA or in IL-IRA overexpression models, and similar to humans, mice that lack IL-1RA display arthritis and skin manifestations [63–65]. However, development of some arthropathy, such as bone erosions, was dependent the background strain of mice [63–65].

Similar to CAPS, DIRA patients are routinely administered with the IL-1R antagonist anakinra. Treatment has shown to rapidly resolves all signs of DIRA including severe osteopenia and bone lesions [66].

## Pathological Roles of IL-1 in the Skeleton

It is increasingly clear that dysregulated inflammasome action and subsequent IL-1 secretion have pathological effects on the skeleton (Table 2). Herein, we outline the pathologies associated with aberrant inflammasome/IL-1 family actions with pathologies of bone accrual and loss.

**Table 2:** Summary of Inflammasome/IL-1 bone pathology in small animal model studies

Disease/Pathology	Results in knock-out mice or after inflammasome/IL-1 related therapies	References
Cryopyrin-associated periodic syndromes	• MCC950 treatment in reduced NLRP3 activation and increased body weight and survival in a CAPS mouse model • All NOMID-associated inflammatory symptoms were prevented in mice lacking Gasdermin D	[43] [45•]
Heterotopic ossification	• *Il1r1*^−/−^ mice had reduced neurogenic heterotopic ossification after spinal cord injury • IL-1 therapy (anakinra) abolished heterotopic ossification in a BMP-induced mouse model of FOP	[67•] [68]
Fracture/Bone defect repair	• Treatment with an NLRP3 inhibitor (glyburide) improved healing in a mouse diabetic fracture model • Targeting NLRP3 via short hairpin RNA, improved alveolar bone defect healing in diabetic rats • Fracture healing unchanged in *Il1r1*^−/−^ mice and IL-1β administration did not alter fracture repair	[69] [70] [71]
Rheumatoid arthritis	• IL-1 blockade (anti IL-1α/β and IL-1RA) ameliorates arthritis in CIA mouse model • *Il1ra*^−/−^ develop spontaneous arthritis • Arthritic phenotypes were reversed in *Nlpr3*^−/−^*, casp1/11*^−/−^ and *Il1r1*^−/−^ mice in a spontaneous arthritis mouse model (Myeloid-cell-specific deletion of *A20*) • MCC950 attenuated synovial inflammation in CIA mouse model • IL-18 blockade (IL-18 neutralising antibody, *Il18*^−/−^ and IL-18BP) decreased arthritis severity and cartilage damage in CIA mouse model • Intraarticular overexpression of IL-18 leads to joint inflammation and cartilage damage in mice • IL-1β blockade reduced arthritis severity, but deletion of *Nlrp3* or Nlrc4 genes did not impact arthritis in an AIA mouse model • *Pycard*^−/−^ mice were protected from developing arthritis in CIA mice, but no protection was noted in *Nlpr3*^−/−^ or *Casp1/11*^−/−^ mice	[72] [63] [73] [74•] [75, 76, 77] [78] [79] [80]
Osteoarthritis	• NLRP3 inhibition (CY-09 treatment) attenuated cartilage damage in a meniscectomy OA mouse model • *Il1a*^*−/−*^*, Il1*b^−/−^, and *Nlrp3*^*−/−*^ mice develop OA features (synovial inflammation and cartilage proteoglycan loss) similar to WT mice in a meniscectomy OA mouse model • OA phenotypes were not prevented in *Il1a*^*−/−*^*, Il1b*^*−/−*^, and *Pycard*^−/−^ and *Nlrp3*^*−/−*^ mice in a calcium crystal OA mouse model	[81•] [82] [83]
Osteoporosis	• IL-1RA and IL-18BP administration reduced bone loss in an ovariectomy mouse model • *Nlrp3*^−/−^ mice were protected from excessive bone loss in an ovariectomy mouse model • *Nlrp3*^−/−^ mice were protected from age-related bone loss	[84•, 85] [86•] [87]
Osteolysis	• *Casp1*^−/−^ mice have reduced particle induced osteolysis • IL-1β blockade reduced particle induced osteolysis	[88] [89]
Periodontitis	• Alveolar bone loss was reduced in *Nlpr3*^−/−^ mice and mice treated with MCC950 in a ligature induced periodontitis model • Aged *Nlpr3*^−/−^ mice develop less alveolar bone loss and MCC950 treatment attenuated the severity of periodontitis associated with ageing • Alveolar bone loss was reduced in P.gingivalis challenged *Nlpr3*^−/−^ mice	[90] [91•] [92]

### Bone Accrual Associated Diseases

#### Heterotopic Ossifications

**Fig. 2:**
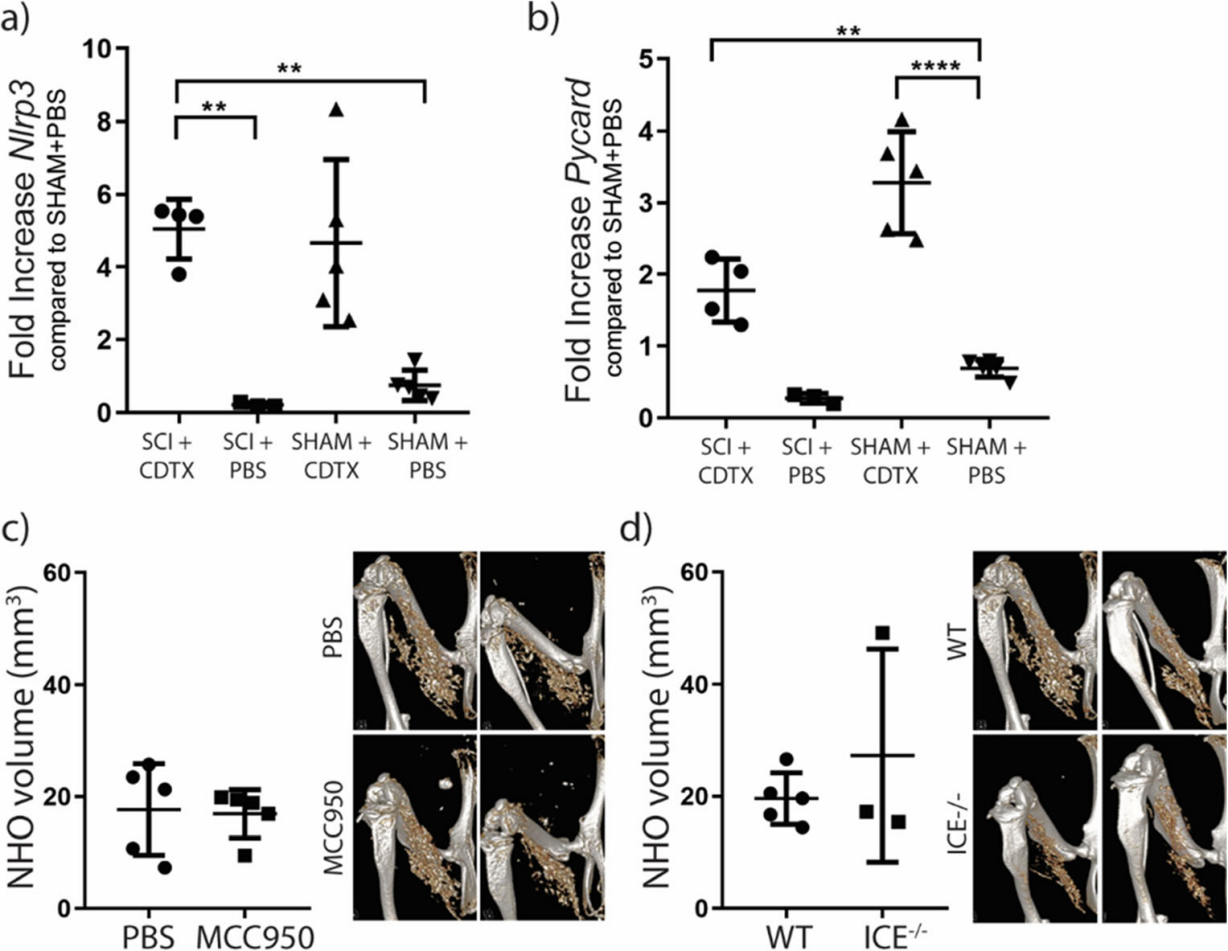
The Inflammasome and NHO pathogenesis. **a**, **b** C57BL/6 mice underwent spinal cord injury (SCI) surgery and muscle injury was induced via an intramuscular injection of CDTX or control PBS injection, or SHAM surgery with either an intramuscular injection of CDTX or PBS injection. Both *Nlrp3* (**a**) (**p = 0.0012, *p* = 0.0015) and *Pycard* (**b**) (**p = 0.0096, ****p < 0.0001) mRNA expression was upregulated in whole muscle by CDTX intramuscular injection on day 4 post-injury. **c** C57BL/6 mice underwent SCI with an intramuscular injection of CDTX and treated with either PBS or MCC950 (20 mg/kg daily ip) for the first 7 days post-surgery. NHO volumes measured by microcomputed tomography (μCT), with two representative images per treatment group. **d** C57BL/6 and ICE^−/−^ mice underwent SCI with an intramuscular injection of CDTX, NHO volumes measured by μCT, with two representative images per group. Each dot represents one mouse; results are presented as mean ± SD, single experiments

Neurogenic heterotopic ossifications (NHO) are bones that develop in periarticular muscles and are a frequent complication after spinal cord injuries (SCI). NHOs frequently lead to ankylosis of the affected joints [93], and cause nerve and blood vessel compression, with treatment limited to surgical resection [94, 95]. Using a mouse model of NHO after SCI, we have established that IL-1 signaling contributes to NHO pathology in mouse and humans [67•]. *Il1r1*^−/−^ mice had significantly reduced NHO development compared to wild-type controls, and IL-1β was significantly enhanced in the plasma of NHO patients and expressed by CD68^+^ macrophages in human NHO biopsies. As previously mentioned, a high proportion of infants with DIRA (unopposed IL-1 signaling) develop periarticular heterotopic ossifications [62]. Given all these observations, we investigated the role of the NLRP3 inflammasome in NHO development. *Nlrp3* and *Pycard* mRNA was significantly upregulated after muscle injury, irrespective of SCI (Fig. 2a, b). Interestingly, there was no change in NHO bone volumes in mice administered MCC950 or in mice defective for both *Casp1* [96] and *Casp11* genes (ICE^−/−^) compared to controls (Fig. 2c, d). Given that NHO bone volumes were not attenuated in *Il1a*^−/−^ or *Il1b*^−/−^ mice [67•], or when components of the inflammasome were inhibited or absent, both our studies suggests that both IL-1 proteins act in concert and specifically targeting the NLRP3 inflammasome or either of the two IL-1 genes separately, is not sufficient to reduce NHO development.

There is also evidence of IL-1-driven pathogenesis in other forms of heterotopic ossification (HO). Increased plasma IL-1β was also noted in an Achilles’ tenotomy and dorsal burn injury mouse model of trauma-induced HO [97]. Fibrodysplasia ossificans progressiva (FOP) is a rare autosomal genetic disease caused by dominant gain-of-function mutations in the coding sequence of the *ACVR1* gene, encoding the activin A receptor type 1, a bone morphogenetic protein (BMP) type I receptor [98]. FOP children develop soft tissue swellings early in life and develop progressive HO which cover the whole body becoming ultimately fatal. In a BMP-induced mouse model of HO, anti-IL-1 therapy abolished HO development [68]. In the *Acvr1*^R206H^ mouse model of FOP, IL-1β was detected by immunohistochemistry at higher levels in *Acvr1*^R206H/+^ lesions compared to wildtype [99]. Anakinra has also been administered to FOP patients. During ‘flare-ups,’ IL-1β plasma levels increased, which reduced after anakinra [100]; however, there were insufficient data to determine how important IL-1 therapy was in comparison to therapies targeting other inflammatory cascades.

#### Fracture and Bone Defect Healing

Diabetics are known to have increased fracture risk and impaired bone healing, and the inflammasome has been shown to play a role in fracture repair in this context. In a mouse model of diabetic-induced fracture, treatment with the NLRP3 inflammasome inhibitor glyburide reduced pro-inflammatory cytokine expression, fracture-associated osteoclasts and also increased callus volume and maximum torque and yield torque [69]. Targeting NLPR3 via short hairpin RNA also improved alveolar bone defect healing in diabetic rats [70].

In other non-diabetic fracture models, IL-1β was upregulated during early phases of bone repair [71], and a role for IL-1β in hematoma fibrin clots has been proposed [101]. IL-1β administration in a rat tibial bone defect model significantly increased osteoblasts number at the defect site [102]; however, there was no change in callus size, cartilage volume, and bone volume in *Il1r1*^−/−^ mice [71]. Finally, repeated doses of IL-1β after fracture had no impact on healing, suggesting a minor or a at least a redundant role for IL-1 in non-diabetic fracture healing.

#### Ankylosing Spondylitis

Ankylosing spondylitis (AS) is a chronic autoinflammatory disease affecting the axial skeleton including the spine and sacroiliac joints, where between 1 and 33% of patients develop syndesmophytes (bony spurs occurring at vertebral corners that potentially fuse the spine) over 2 years of disease progression [103].

Peripheral blood mononuclear cells (PBMC) isolated from AS patients express higher mRNA levels of *NLRP3*, *PYCARD*, *NLRC4*, *CASP1*, and increased serum IL-1β and IL-18 compared to healthy controls [104]. To date, only a few clinical studies on a small number of patients have evaluated the effects of anakinra in managing inflammation in AS. In one open label study, anakinra treatment for 12 weeks significantly decreased non-specific inflammatory measurements, CRP, and erythrocyte sedimentation rate (ESR), and led to a 61% improvement in entheseal lesions at the lumbar spine and sacroiliac joints detected by magnetic resonance imaging [105]. However, in another open label study, anakinra treatment for 24 weeks only led to limited and temporary improvement of clinical symptoms for a small subset of patients [106].

Overall, the above studies demonstrated that the inflammasome and IL-1β are activated in AS patients; however, to date there is lack of evidence for long-term therapeutic benefit of inflammasome/IL-1 inhibition. Moreover, the effects of targeting inflammasome/ IL-1 on osteoproliferation in AS has not been reported in either animal models or AS patients.

#### Osteoarthritis

Osteoarthritis is a progressive disease featured by cartilage degeneration, subchondral bone modification, crystal deposition, and osteophyte formation [107]. Inflammatory cytokines and catabolic enzymes such as matrix metalloproteinases are associated with synovial inflammation and cartilage degeneration [107].

Among the current OA rodent models, upregulation of NLRP3, caspase-1, GSDMD, IL-1β, and IL-18 had been reported at mRNA and protein level in OA models induced by mechanical stress (meniscectomy) [81, 82] or enzymatic degradation (intraarticular injection of papain) [108]. However, NLRP3 blockade and gene deficiency have shown inconsistent results in OA models. For example, treatment with the NLRP3 inhibitor CY-09, decreased *Nlrp3* and *Gsdmd* expression, and attenuated cartilage damage in the meniscectomy induced OA [81•]. However, *Il1b*^−/−^ or *Nlrp3*^−/−^ mice were not protected from cartilage degeneration and osteophyte formation after meniscectomy [82]. Micro crystal deposition is commonly found in the synovium. In a mouse model of OA (induced by intraarticular injection of basic calcium phosphates, the primary crystal found in OA cartilage), OA phenotypes were not prevented in *Il1a*^−/−^, *Il1b*^−/−^, *Pycard*^−/−^, or *Nlrp3*^−/−^mice [83]. Intraarticular injection of IL-1β suppressed proteoglycan synthesis and enhanced proteoglycan breakdown in a mouse OA model suggesting IL-1β can induce cartilage degeneration [109].

While OA patient synovial tissue and cartilage biopsies display enhanced IL-1β, IL-18, and caspase-1 expression [110, 111], inflammasome and IL-1 therapies for prevention of cartilage damage or osteophyte formation has not been investigated. The effect of IL-1 blockade (anakinra or AMG108, a humanized monoclonal antibody to IL1R1) on pain relief has been investigated but with disappointing results [112, 113].

### Bone Loss Associated Diseases

#### Inflammatory Arthritis

The term inflammatory arthritis is used to describe a group of autoinflammatory disorders, one of the most recognized is rheumatoid arthritis (RA). RA is an autoimmune inflammatory disease characterized by synovial inflammation, cartilage, and bone erosion which leads to progressive joint deformation [114]. Various pathways have already been identified in promoting RA pathogenesis [115•].

Preclinical models in small animals have been vital in establishing pathological roles for the inflammasome, IL-1β, and IL-18 in RA. Targeting IL1R signaling by administrating anti-IL1α/β or IL1RA ameliorates arthritis in the collagen-induced arthritis (CIA) mouse model [72]. Conversely, unopposed IL-1 signaling, due to the deletion of the *Il1ra* gene, led to development of spontaneous arthritis [63]. Spontaneous arthritis induced by myeloid-specific deletion of the RA susceptibility gene *Tnfaip3* requires inflammasome signaling, as a deficiency in either *Nlrp3*, *casp1/11*, or *Il1r1* genes in this model rescued arthritic phenotypes [73]. Treatment of CIA mice with the NLRP3 inhibitor MCC950 also attenuated synovial inflammation and bone erosion [74•]. IL-18 expression is also significantly upregulated in the CIA mouse model [75]. Targeting IL-18 via a neutralizing antibody, IL-18 binding protein (IL18BP) or using *Il18*^−/−^ mice all significantly decreased the severity of arthritis and cartilage damage [75–77]. Conversely, intraarticular overexpression of IL-18 leads to joint inflammation and cartilage damage [78].

NLRP3-independent pathways also participate in RA pathogenesis. IL-1β blockade significantly reduced arthritis severity in an antigen-induced arthritis (AIA) mouse model, while *Nlrp3* or *Nlrc4* gene deletion did not affect AIA [79]. Deletion of the *Pycard* gene (encoding ASC) protected CIA mice from developing arthritis and bone erosion showing the importance of canonical inflammasomes; however, *Nlrp3* and *Casp1* null mice developed comparable disease to WT mice [80]. Overall, these studies suggest both NLRP3-dependent and independent pathways (via other canonical inflammasomes) could potentially contribute to IL-18 and IL-1β pathologies in preclinical RA models.

Recent studies have documented an active inflammasome in RA patients. Total leukocytes from RA patients in the acute phase of disease expressed higher levels of ASC, active caspase 1 and NLRP3 compared to healthy controls, and in vitro stimulation of total leukocytes resulted in enhanced IL-1β secretion [116]. IL-18, NLRP3 expression, and caspase-1 activity were also upregulated in RA synovial tissue compared to osteoarthritis (OA) patients [74, 117]. In vitro stimulation of RA synovial fibroblasts with IL-18 increased the production of the chemokines IL-8/CXCL8, epithelial-neutrophil activating protein/CXCL5, growth-regulated oncogene α (GROα)/CXCL1, and the CC chemokine; CCL2 [118] as well as the angiogenic factors stromal cell-derived factor 1α (SDF-1α/CXCL12) and vascular endothelial growth factor A (VEGF-A) [119].

Anakinra was approved for patients with moderate-severe RA who are unresponsive to initial disease-modifying antirheumatic drugs in 2009–2010. Anakinra improved clinical arthritic symptoms and ameliorated radiographic progression [120]; however, anakinra was less effective than the TNF-α antagonist etanercept, with a higher incidence of adverse events [121, 122] and FDA approval was removed. Nevertheless, anti-IL-1 therapy was recommended in RA patients with type 2 diabetes in the TRACK (Treatment of Rheumatoid Arthritis and Comorbidities with Kineret) study as anakinra as it was as efficacious as TNF inhibitors, and enabled a decrease in antidiabetic and glucocorticoid therapies which was not observed in patients treated with TNF inhibitors [123].

#### Osteoporosis

Osteoporosis is characterized by low bone mass and deterioration of bone architecture, often associated with aging, reduced female hormones due to menopause, and metabolic diseases which disturbs bone remodeling [124].

IL-1β and IL-18 contribute to pathological bone loss in osteoporotic mouse and rat models. Ovariectomized (Ovx) mice mimic postmenopausal-induced bone loss, which can be arrested via IL-1Ra [125, 126] and IL-18BP [84•] administration. *Nlrp3* deficiency also significantly slows down femoral bone loss [87]. Bone loss in mouse models of ovariectomy, continuous parathyroid hormone (PTH) infusion, or RANKL-induced osteopenia was also significantly improved in *Nlrp3*^−/−^ mice [86•].

Studies have demonstrated an association between IL-1β, IL-18, and postmenopausal bone loss in humans. In vitro stimulation of monocytes isolated from healthy premenopausal, untreated healthy postmenopausal, estrogen-treated postmenopausal, and hormone-treated postmenopausal women with osteoporosis showed that menopause significantly increased the capacity of monocytes to produce IL-1β, which was reversed by ovarian hormones [127]. In vitro stimulation of whole blood cells harvested from postmenopausal osteoporosis patients produced higher levels of IL-1β, IL-6, TNF-α, IFN-γ, and GM-CSF compared to healthy controls, and IL-1β levels were negatively correlated with lumbar spine (L2-4) bone mineral density in patients [127, 128]. Moreover, reduction of IL-18BP in PBMCs isolated from osteoporotic patients indicated increased IL-18 signaling in osteoporosis [84•]. Only one clinical study has evaluated IL-1 therapy for osteoporotic patients. In this study, menopausal women were treated with anakinra for 3 weeks without hormone treatment. Serum bone resorption markers were significantly increased in saline-treated patients while anakinra moderately dampened this trend but this was not statistically significant [129]. Therefore, despite IL-1 and IL-18 playing pathological roles in osteoporosis, targeting osteoporosis with IL-1-blocking monotherapy may be insufficient to prevent osteoporosis.

#### Osteolysis

Joint replacement surgery is an effective way to restore mobility and relieve pain resulting from arthritis, fractures, and avascular necrosis. However, periprosthetic osteolysis, which involves focal bone erosion around implants, is the leading cause of joint replacement failure [130•].

Studies show an increased frequency of IL-1α and IL-1β expressing cells in the pseudo-synovial membrane of the prostheses compared to non-arthritic samples [131]. Serum IL-1β is also associated with early aseptic loosening of hip prosthesis [132]. Orthopedic wear debris has been suggested to initiate inflammation and subsequent osteolysis. In vitro studies demonstrated that metal wear particle debris increase the secretion of IL-1β and IL-18 in mouse bone marrow-derived macrophages [88, 89], which was reduced by inhibiting or knocking-down NLRP3 and caspase-1 [133]. Poly(methyl methacrylate) (PPMA) debris also enhance osteoclast differentiation in vitro [134], and bone marrow derived osteoclast precursors from *Nlrp3*^−/−^ and *Il1r1*^−/−^ mice showed less differentiation after exposure to bone particles [86, 90]. Moreover, implantation of PPMA, bone cement, or metal debris in calvaria induced significant bone loss, and *Casp1* deficiency or anti-IL-1β treatment significantly ameliorated particles-induced osteolysis [88, 89]. These lines of evidence suggest that inflammasome signaling plays an important role in periprosthetic osteolysis.

#### Periodontitis

Periodontitis is a disease characterized by inflammation of the tissues surrounding teeth, and is frequently associated with poor oral hygiene. Mouse models of periodontitis were utilized to investigate the role of the inflammasome in periodontal pathology. In a model of tooth ligature-induced periodontitis, *Nlrp3*^−/−^ mice or mice treated with MCC950 had reduced alveolar bone loss measured by μCT compared to control mice and reduced in vitro osteoclast differentiation [90]. As periodontal diseases are more prevalent in older individuals, one study illustrated that serum IL-1β levels increased with age and were directly proportional to the extent of periodontal destruction. Aged *Nlrp3*^−/−^ mice developed less alveolar bone loss compared to wild-type mice and continuous administration of MCC950 attenuated the severity of the bone loss, suggesting that NLRP3 inflammasome contributes to periodontitis progression with aging [91•].

Imbalances of the oral microbiome often lead to oral dysbiosis [135], which is a contributing factor to periodontitis. *Nlrp3*^−/−^ mice challenged with live *Porphyromonas gingivalis* (*P*. *gingivalis*), a Gram-negative oral commensal bacterium, had significantly reduced bone loss after 18 weeks, compared to wild-type mice. Additionally, wild-type mice challenged with *P*. *gingivalis* had elevated expression of *Il1b* and *Il18* mRNA in the periodontium [92], which was also noted by others [136]. Bruton’s tyrosine kinase (BTK) is already known to regulate osteoclast maturation and fusion [137, 138], and was shown to also modulate the NLRP3 inflammasome, through interaction with ASC and NLRP3 [139]. Administration of Acalabrutinib a BTK inhibitor, has recently been shown to reduce alveolar bone loss in a *P*. *gingivalis* LPS-induced periodontitis mouse model [140]. Whether BTK blockade also impacts inflammasome-driven bone loss remains to be investigated.

In humans, single-nucleotide polymorphisms of the *IL1B* gene increase susceptibility to periodontitis [141], and gingival tissues from periodontitis patients show elevated mRNA expression of *NLRP3* and *IL1B* genes [142]. MCC950 treatment of human periodontal cells in vitro suppressed LPS-dependent cell death and IL-18 and IL-1β secretion [143]. MCC950 also significantly reduced expression of NLRP3 and Caspase-1 in cell lysates from human periodontal ligament cells [143]. A recent clinical study reported that patients with periodontitis show elevated serum and saliva NLRP3 concentrations [144], and elevated NLRP3, ASC, and IL-1β concentrations in saliva are proposed biomarkers for periodontal clinical status [145]. These accumulating lines of evidence suggest the inflammasome play important roles in periodontitis.

## Conclusions

Overall, mounting evidence links the inflammasome/IL1 family activity to bone pathologies. To date, these pathologies are treated by blocking IL-1/IL-1R. While IL-1-blocking therapies can alleviate disease severity, chronic IL-1 blockade can also lead to an increased risk of infections and sepsis, which is why selective targeting of inflammasomes is an attractive future therapeutic strategy. Therapeutic challenges may arise due to redundancies in proteolytic cleavage pathways as well as redundancy of IL-1 isoform signaling. However, there are promising developments in inflammasome therapeutics [146], and new inflammasome/IL-1 based therapies are already in clinical trials for CAPS, RA, and OA (Table 3). There is also ongoing development of new inflammasome small molecule inhibitors, as well as targeting post transcriptional and translational modifications of inflammasomes, all of which will allow for novel therapeutic avenues for managing inflammasome-driven diseases.

**Table 3:** Inflammasome/IL-1 therapeutics in clinical trials for bone related diseases

Target	Disease	Therapeutic	Clinical trial/s and reference
NLRP3	RA	MCC950	Phase II – discontinued due to liver toxicity [147]
	OA	OLT1177	Phase II (NCT01768975) [148] Phase II (NCT02104050)
	CAPS	IZD334 Inzomelid	Phase I (NCT04086602) [149] Phase I (NCT04015076)
IL-1β	RA	Canakinumab	Phase II (NCT00554606)
	CAPS	Canakinumab	Phase III (NCT00685373, NCT01576367, NCT01302860) Phase III (NCT00770601, NCT01105507)
IL1R1	RA	Anakinra	Completed [120]
